# Sensing the Messenger: Potential Roles of Cyclic-di-GMP in Rickettsial Pathogenesis

**DOI:** 10.3390/ijms23073853

**Published:** 2022-03-31

**Authors:** Hema P. Narra, Abha Sahni, Krishna Mohan Sepuru, Jessica Alsing, Sanjeev K. Sahni

**Affiliations:** 1 Department of Pathology, Institute for Human Infections and Immunity, University of Texas Medical Branch, Galveston, TX 77555, USA; absahni@utmb.edu (A.S.); jealsing@utmb.edu (J.A.); 2 Department of Molecular Biosciences, Howard Hughes Medical Institute, The University of Texas at Austin, Austin, TX 78712, USA; krishna.sepuru@austin.utexas.edu

**Keywords:** *Rickettsia*, response regulator PleD, cyclic di-GMP, host-pathogen interaction, virulence

## Abstract

Pathogenic bacteria causing human rickettsioses, transmitted in nature by arthropod vectors, primarily infect vascular endothelial cells lining the blood vessels, resulting in ‘endothelial activation’ and onset of innate immune responses. Nucleotide second messengers are long presumed to be the stimulators of type I interferons, of which bacterial cyclic-di-GMP (c-di-GMP) has been implicated in multiple signaling pathways governing communication with other bacteria and host cells, yet its importance in the context of rickettsial interactions with the host has not been investigated. Here, we report that all rickettsial genomes encode a putative diguanylate cyclase *pleD*, responsible for the synthesis of c-di-GMP. In silico analysis suggests that although the domain architecture of PleD is apparently well-conserved among different rickettsiae, the protein composition and sequences likely vary. Interestingly, cloning and sequencing of the *pleD* gene from virulent (Sheila Smith) and avirulent (Iowa) strains of *R. rickettsii* reveals a nonsynonymous substitution, resulting in an amino acid change (methionine to isoleucine) at position 236. Additionally, a previously reported 5-bp insertion in the genomic sequence coding for *pleD* (NCBI accession: NC_009882) was not present in the sequence of our cloned *pleD* from *R. rickettsii* strain Sheila Smith. In vitro infection of HMECs with *R. rickettsii* (Sheila Smith), but not *R. rickettsii* (Iowa), resulted in dynamic changes in the levels of *pleD* up to 24 h post-infection. These findings thus provide the first evidence for the potentially important role(s) of c-di-GMP in the determination of host-cell responses to pathogenic rickettsiae. Further studies into molecular mechanisms through which rickettsial c-di-GMP might regulate pathogen virulence and host responses should uncover the contributions of this versatile bacterial second messenger in disease pathogenesis and immunity to human rickettsioses.

## 1. Introduction

The genus *Rickettsia*, in the family Rickettsiaceae of the order Rickettsiales, now encompasses an expanding number of Gram-negative, intracellular α-proteobacteria distributed across the globe and transmitted to humans as accidental hosts by arthropod vectors, including fleas, lice, mites, and ticks. The most prominent pathogenic species causing vector-borne rickettsioses belong to the spotted-fever group represented by *R. rickettsii* and *R. conorii*, and typhus group, including *R. prowazekii* and *R. typhi* [1,2]. As endotheliotropic pathogens, rickettsiae preferentially infect endothelial cells lining the small- and medium-sized vessels, resulting in systemic inflammation and compromised barrier integrity as the major pathophysiological sequelae, leading to increased vascular permeability and tissue edema in vital organs such as the lungs and brain [3,4]. Consequently, host–pathogen interactions with a focus on endothelial responses and vascular dysfunction or damage constitute an important area of study to achieve a comprehensive mechanistic understanding of rickettsial pathogenesis and immunity. Not surprisingly, a majority of endothelial responses to disseminated infection with rickettsiae are mediated through the activation of cellular regulators, including nuclear transcription factor-kappa B (NF-kB) and interferon regulatory factors [5,6,7]. In this regard, our prior findings have documented induction of a type I interferon (IFN) response by cultured human microvascular endothelial cells (HMECs) infected with *R. conorii* and its involvement in the autocrine and/or paracrine activation of the Janus kinase-signal transducer and activator of the transcription (JAK-STAT) signaling pathway, and increased expression of a number of downstream IFN-stimulated innate-response genes [8,9].

Cyclic dinucleotides are now well-appreciated to manifest important roles in the determination of host innate immune responses to bacterial and viral infections. Bacteria produce cyclic diguanylate monophosphate (c-di-GMP), cyclic diadenylate monophosphate (c-di-AMP), and cyclic guanosine monophosphate-adenosine monophosphate (c-GAMP) as second messengers for the fine regulation of a variety of cellular processes [10,11]. Of these, c-di-GMP synthesized from two GTP molecules by diguanylate cyclases (DGCs) has been determined to be ubiquitously utilized by bacteria for the governance of numerous life-cycle activities, including biofilm formation, motility, pathogenesis, and virulence [11,12,13]. The catabolism of c-di-GMP into linear 5-phosphoguanylyl-(3-5)-guanosine (pGpG) or GMP occurs through phosphodiesterase activities (PDEs). Importantly, catalytic domains of DGCs and PDEs, named as GGDEF and EAL or HD-GYP, respectively, have been extensively characterized biochemically, structurally, and genetically to suggest a high degree of conservation with an almost ubiquitous presence in bacteria [14,15]. The active site of the GGDEF domain is involved in GTP binding, resulting in the synthesis of c-di-GMP, which is then recognized by the mammalian innate immune system to trigger host type I interferon responses [16,17]. The ubiquitous effects of c-di-GMP on the survival and virulence strategies of most bacterial species, in conjunction with the lack of both synthetic and degradative enzymes in all other domains of life, make this bacterial messenger a promising candidate for the development of immune-based adjuvants or therapeutics [14].

Despite the tendency for evolution by genomic reduction, the genomes of *Rickettsia* species carry *pleD* predicted to encode for a putative diguanylate cyclase responsible for the synthesis of c-di-GMP. However, the expression of this important gene in target host cells and its functional roles in rickettsial pathogenesis remain an open question. As the critical first step to address this important knowledge gap, the aims of this study focus upon the demonstration of the presence of *pleD* in the genomes of different *Rickettsia* species, characterization of its domain architecture in well-characterized strains of varying virulence, and determination of its expression profile during rickettsial infection of the host microvascular endothelium as the preferred target niche.

## 2. Results

### 2.1. PleD Is Present and Conserved among Rickettsia Species

**Figure 1:**
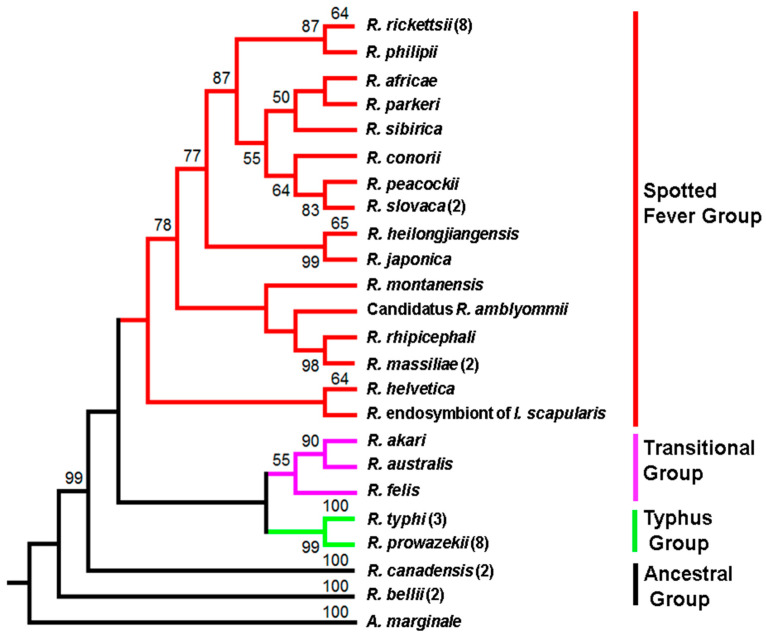
Phylogeny of *Rickettsia* species inferred from PleD protein sequences. The numbers above the branches indicate bootstrap support values. The PleD sequence of *A. marginale* str. Florida was used as an outgroup. The number in parenthesis indicates the number of strains used in the analysis. Branch color represents *Rickettsia* species belonging to different groups. Red: spotted fever group, Purple: Transitional group, Green: Typhus group, and Black: Ancestral group.

A phylogenetic tree based on amino-acid composition of PleD from representative species of all major subgroups, i.e., ancestral, spotted fever, typhus, and translational, illustrates its presence and conservation in the Genus *Rickettsia*. The protein with a predicted approximate molecular mass of ~52 kDa is encoded by a nucleotide sequence of 1353 bases and is comprised of 450 amino acids. As shown in Figure 1, the phylogeny is based upon protein sequences for 16 different species from the spotted fever group, 3 from the transitional group, and 2 each from the typhus and ancestral group. For spotted fever species, namely *R. rickettsii*, *R. massiliae*, and *R. slovaca*, both major prototypical representatives of the typhus group *R. prowazekii* and *R. typhi*, and *R. bellii* and *R. canadensis* of the ancestral group, different specific strains for which the genomes have been sequenced and annotated were also included.

### 2.2. Preservation of Domain Organization of PleD in Rickettsia Species

**Figure 2:**
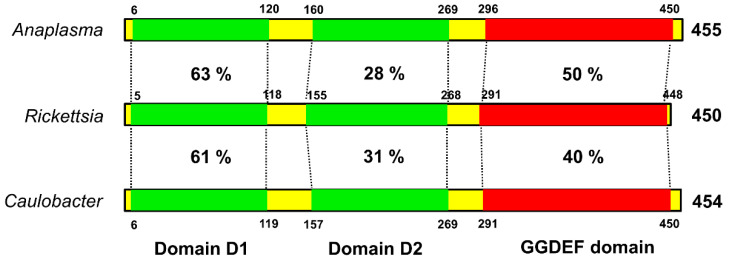
The conserved domain architecture of rickettsial PleD. The rickettsial PleD domains (receiver domain 1, adapter domain 2, and GGDEF domain) were identified using blastp and compared with their counterparts in *Anaplasma* and *Caulobacter.* The receiver domain 1 and GGDEF domain of *Rickettsia* showed higher sequence identity with those present in *Anaplasma* and *Caulobacter*, whereas the adapter domain 2 is relatively less conserved. Sequence identity is presented as percentage. The numbers above/below the bars correspond to domain coordinates.

The biological importance of c-di-GMP as a bacterial second messenger is underscored by the omnipresence of the diguanylate cyclase (DGC) domain (also referred to as GGDEF or DUF1) in bacterial genomes. Structurally, PleD is composed of a CheY-like receiver domain (D1), a CheY-like adapter domain (D2), and a DGC domain. The D1 domain contains conserved residues, acts as a phosphoacceptor, and is involved in activation of the response regulator. The D2 domain is involved in dimerazation and binding to cyclic di-GMP at the DGC-D2 interface, leading to structural stabilization and prevention of catalysis [18]. Despite nearly ubiquitous distribution and documented regulatory relevance of DGC proteins in bacteria, structural and functional information about this class of regulators in *Rickettsia* species is largely missing. To address this, we performed the amino-acid sequence (blastp) comparison of the PleD domains of *Rickettsia* with the corresponding domains of a pathogenic α-proteobacterium *Anaplasma phagocytophilum* and a free-living, dimorphic α-proteobacterium *Caulobacter crescentus*. Such analysis revealed the highest level of conservation in D1 as suggested by a homology of 63 and 61%, respectively, with *Anaplasma* and *Caulobacter*. A similar comparison for the catalytic GGDEF domain suggested identity levels of 50% between rickettsial and *Anaplasma* PleD, and 40% between rickettsial and *Caulobacter* PleD, while adapter domain D2 displayed significant divergence among these bacteria (Figure 2).

### 2.3. Sequencing and Comparison of pleD from Virulent and Avirulent Strains of R. rickettsii

**Figure 3:**
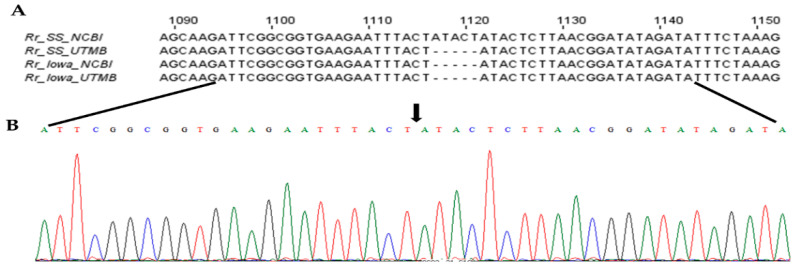
The nucleotide sequence and chromatogram showing the absence of 5-base insertion in the *pleD* gene of *R. rickettsii* str. Sheila Smith. The full-length *pleD* from *R. rickettsii* strains Sheila Smith (Rr_SS_UTMB) and Iowa (Rr_Iowa_UTMB) were sequenced and aligned with sequences available in NCBI (Rr_SS_NCBI and Rr_Iowa_NCBI). The sequence alignment (**A**) and chromatogram (**B**) show the absence (shown by black arrow) of ‘ATACT’ indel in *pleD* sequence of *R. rickettsii* str. Sheila Smith.

We next set out to investigate the possibility of PleD’s contributions to the host-cell responses to infection and a potential virulence factor of rickettsiae. Differences in the pathogenicity and virulence of different strains of spotted-fever group *Rickettsia* species, including *R. rickettsii*, are well established [3,19]. In a previously published study, application of bioinformatics-based strategies to define the biological basis of variations in virulence revealed a number of single-nucleotide polymorphisms, insertions, and deletions unique to specific strains and capable of distinguishing between them to allow for the distinction between virulent and avirulent *R. rickettsii* [20]. Intriguingly, our database search for the genomic sequences of *pleD* in a highly virulent Sheila Smith strain vis-à-vis an avirulent Iowa strain of *R. rickettsii* resulted in the identification of a fully annotated *pleD* in Iowa, but not in *R. rickettsii* Sheila Smith, and alignment of published sequences (NCBI Accession numbers NC_009882 (Sheila Smith) and NC_010263 (Iowa)) suggested a 5-bp insertion (ATACT) at positions 1117 to 1121, with respect to the start site, of the *pleD* gene in *R. rickettsii* Sheila Smith. To resolve this possible ambiguity, we cloned and sequenced *pleD* from both strains to demonstrate the absence of 5-bp insertion in *R. rickettsii* Sheila Smith and a 100% match between the sequences from Iowa and Sheila Smith strains of *R. rickettsii* (Figure 3; Supplementary File S1).

### 2.4. A Nonsynonymous Substitution in the Nucleotide Sequence Results in an Amino-Acid Change at Position 236 of PleD in Different Strains of R. rickettsii

**Figure 4:**
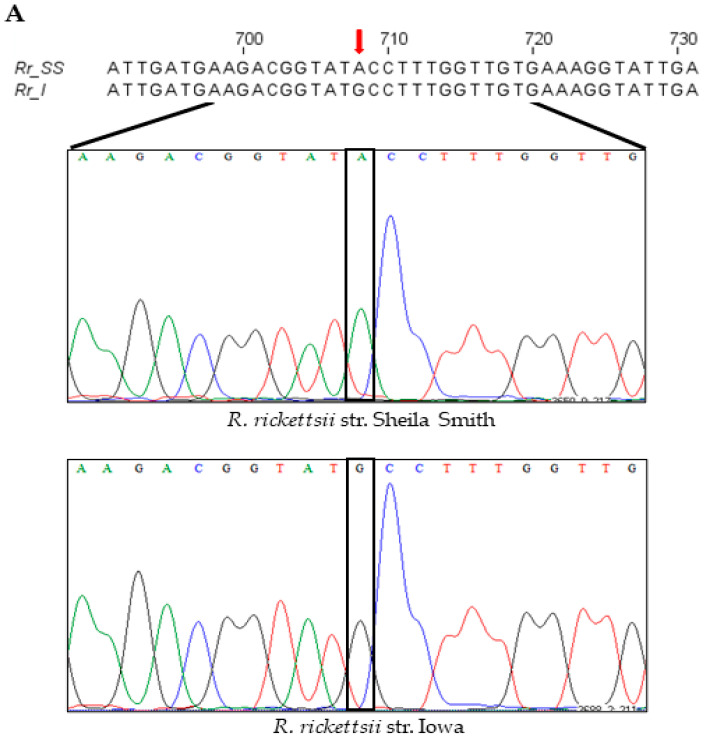

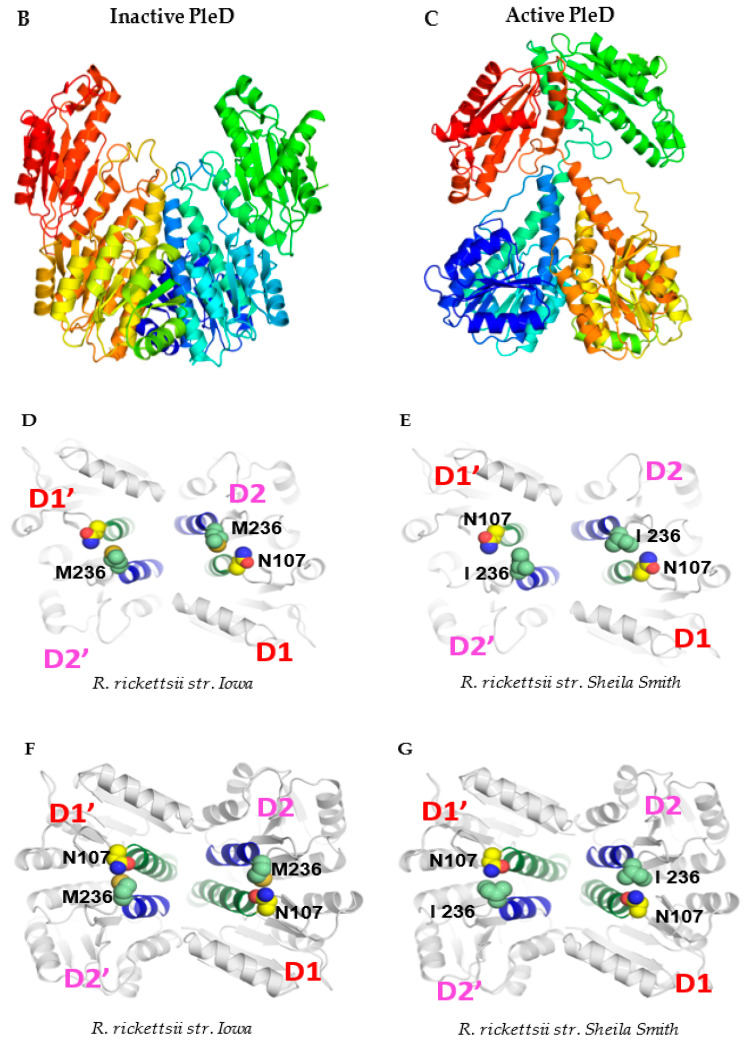
A single-nucleotide polymorphism (SNP) at position 708, with respect to the gene start site, in the *pleD* of *R. rickettsii* str. Sheila Smith and Iowa results in an amino-acid change at a key residue of the protein. (**A**) Nucleotide sequence and chromatograms of *R. rickettsii* str. Sheila Smith and Iowa showing SNP (red arrow) at position 708 with respect to the gene start site. Ribbon representation of the dimeric model structures (perpendicular to the two-fold axis of the stem) of the inactive (**B**) and active (**C**) PleD. In panels (**D**–**G**), the views of the PleD inactive and active dimeric models are rotated by 90° around a horizontal axis with respect to the top panels (**B**,**C**), and shown is the bottom view of the (D1/D2) dimer stem with the diguanylate cyclase (DGC) domain in the rear hide for clarity. The residue N107 (yellow) on receiver domain D1 and M236/I236 (light green) on adaptor domain D2 are highlighted. The α-helix5 on receiver domain and α-helix4 on adaptor domain are colored in green and blue, respectively.

An intriguing and serendipitous finding emerging from the cloning, sequencing, and comparison of the composition of *pleD* from virulent Sheila Smith and avirulent Iowa strains of *R. rickettsii* was the identification of a single-nucleotide substitution (A→G; ‘A’ in Sheila Smith to ‘G’ in Iowa) at position 708 in the coding sequence (Figure 4A). This nucleotide change led to a nonsynonymous substitution of the amino acid at position 236 in the resultant protein, which accordingly was determined to be an isoleucine in strain Sheila Smith versus a methionine in Iowa (Supplementary File S1). In consideration of the structural and functional implications of nonsynonymous mutations due to variations in amino-acid sequences, we next performed a predictive analysis of tertiary structures using the MODELLER program [21] and generated the inactive and active models of encoded PleD proteins from both Iowa and Sheila Smith strains. As expected, the nonactivated PleD formed a weak dimer-interface interaction, while the activated PleD exhibited a strong dimer-interface interaction of the D1 and D2 domains (Figure 4B,C). Despite exhibiting mostly similar structural conformations in both active and inactive forms, the local interactions involving the receiver domain D1 and adaptor domain D2 domains were slightly different between the strains (Figure 4D–G). Interestingly, we identified that the amino acid at position 236 present in D2 is a predicted key residue which is involved in forming an interdomain bridge with asparagine at position 106 present in D1 domain. Importantly, the nature of the interactions between N106-M236 in the PleD of Iowa strain varies compared to the interaction between N106-I236 in the Sheila Smith strain potentially enhancing the stability of the active form of PleD in the virulent Sheila Smith strain.

### 2.5. Differences in pleD Expression Pattern during Infection of Host Endothelial Cells with Virulent and Avirulent Strains of R. rickettsii

**Figure 5:**
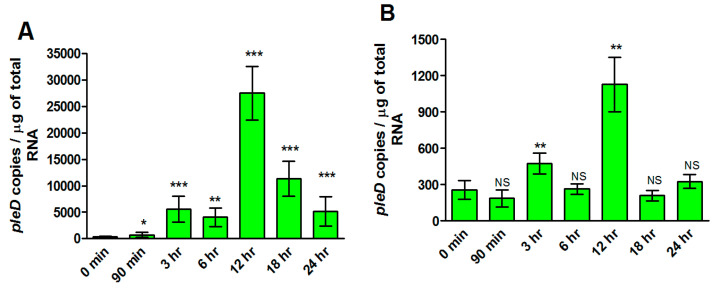
Absolute quantification of *pleD* expression in *R. rickettsii* str. Sheila Smith and Iowa during the infection of host endothelial cells, in vitro. Significant differences in *pleD* expression levels are observed in host cells infected with *R. rickettsii* str. Sheila Smith (**A**) versus *R. rickettsii* str. Iowa (**B**) strain. Highest expression of *pleD* is observed in both *R. rickettsii* str. Sheila Smith and *R. rickettsii* str. Iowa at 12 h post-infection when compared to the control (0 min). * *p* ≤ 0.05, ** *p* ≤ 0.01, *** *p* ≤ 0.001, not significant (NS) *p* > 0.05.

We finally determined expression profiles of rickettsial *pleD* in human microvascular endothelial cells (ECs) infected with *R. rickettsii* strains (MOI = 20) at opposite ends of virulence spectrum. We quantified the bacterial load using citrate synthase-based qPCR assay to ensure that both strains had similar levels of infection at all time points. No significant difference in rickettsial copy number was observed between virulent Sheila Smith and avirulent Iowa strains (Supplementary File S2). Interestingly, *pleD* expression was clearly evident and significantly higher as compared to the basal levels (at 0 h) at all studied timepoints up to 24 h post-infection in ECs infected with the virulent Sheila Smith strain. In contrast, avirulent Iowa triggered such a response of much lower magnitude with significant changes compared to the baseline only at 3 and 12 h post-infection (Figure 5). Absolute quantitation of *pleD* copy numbers further suggested a robust expression of the transcript during infection with virulent *R. rickettsii*, as evidenced by an increase of greater than 10- and 25-fold at 3 and 12 h post-infection when compared to host cells concurrently infected with the avirulent strain.

## 3. Discussion

The diguanylate cyclase and phosphodiesterase groups of enzymes are important signal-transduction proteins involved in the regulation of some critically important attributes of bacterial pathogens via the effector molecule cyclic-di-GMP. Although contributions of c-di-GMP in the governance of processes determining the pathogenesis of many bacteria, such as biofilm formation, motility, and virulence gene expression are now well-established and documented, its biosynthesis and functional roles in Rickettsiales are generally poorly characterized and understood. It is important to acknowledge, however, a seminal investigation in this context by Lai et. al. [22], documenting the presence of a functional PleC histidine kinase and PleD diguanylate cyclase two-component system in *Anaplasma phagocytophilum*, another obligate intracellular bacterium which belongs to the family Anaplasmataceae of the order Rickettsiales and causes granulocytic anaplasmosis in humans. A rather intriguing finding of this study is the inhibitory effect of a hydrophobic c-di-GMP derivative, namely 2′-O-di(tert-butyldimethylsilyl)-c-di-GMP, on the infection of HL60 promyelocytic cells by *A. phagocytophilum*, demonstrating the potential involvement of a c-di-GMP-receptor complex in the regulation of intracellular *A. phagocytophilum* infection [22]. Based upon the conservation of PleD as a single GGDEF domain containing protein among different genera in the family Anaplasmataceae, analysis of cyclic-di-GMP signaling in *Ehrlichia chaffeensis* further suggests vital roles in its proliferation, maturation, and release from host cells with particular emphasis on the dispersion from morula and intracellular movement of bacteria [23]. The present study demonstrates the presence and conservation of PleD among all major subgroups and species of Rickettsiaceae, including well-known pathogens in the spotted fever and typhus groups, which are unique among bacteria due to their propensity to free themselves in the cytoplasm, in contrast to *Anaplasma* and *Ehrlichia*, which remain confined to membrane-bound vacuolar compartments in the host cell.

PleD is a multidomain protein with two N-terminal receiver domains arranged in tandem and a C-terminal GGDEF domain, which is highly conserved in many bacterial species. Due to its association with a variety of domains, it is involved in the sensing of signals in the periplasm, membrane, or cytoplasm. Another remarkable feature of proteins carrying a GGDEF domain is their presence in most bacteria, but absence in archaea and eukaryotes. Thus, it is not surprising that rickettsial GGDEF is seemingly relatively conserved when compared to that of *A. phagocytophilum* and *C. crescentus*, a free-living unicellular bacterium that transitions between an adhesive, sessile form and a motile, planktonic stage as part of its life cycle [24]. Although a similar trend of conservation for N-terminal receiver domain 1 is also evident, domain 2 of rickettsial PleD seems to diverge from that of *Anaplasma* and *Caulobacter*. Whether or not variations in host–pathogen interplay and life-style features might explain the biological basis of these similarities and differences remains an open area of inquiry to be pursued in further detail.

PleD is known to be active in a dimeric form. For instance, dimerization of PleD is required for optimal diguanylate cyclase activity and for sequestering the active form to the pole during differentiation in *Caulobacter* [25]. Based on structural analysis, we have predicted dimerization of PleD protein present in both *R. rickettsii* strains. Additionally, three key interactions between residues E100-R263; N106-M236; R114-D249 have been identified between the receiver domain D1 and adaptor domain D2. These interactions are known to act like a pivot for the transition of the PleD protein from inactive to active form. The nonsynonymous mutation resulting in an amino-acid change at position 236 (isoleucine in strain Sheila Smith versus a methionine in Iowa) could influence the equilibrium of active–inactive forms, as the hydrophobicity index of isoleucine (4.5) is very high compared to methionine (1.9) [26]. This mutation at the key inter-domain interaction can lead to a stable active form of PleD and result in increased production of cyclic di-guanosine monophosphate (c-di-GMP) in the Sheila Smith strain. However, ongoing functional studies aimed at determining the activity of diguanylate cyclase from both virulent and avirulent *Rickettsia* strains will shed light on the role of this mutation on the dimerization, stability, and catalytic activity of the enzyme.

In conclusion, we report the presence of PleD with conserved domain architecture in all *Rickettsia* species. Sequencing of the *pleD* gene from *R. rickettsii* str. Sheila Smith revealed the absence of a 5 bp indel and established the presence of a full-length gene encoding for a functional protein. Interestingly, a nonsynonymous mutation resulting in an amino-acid change at position 236 was identified in the PleD protein encoded by *R. rickettsii* strs. Sheila Smith (virulent) and Iowa (avirulent), and we postulate that this mutation at a key residue is likely to play a vital role in the stability and activity of the dimeric protein. Additionally, the expression of *pleD* transcripts was significantly different between virulent and avirulent *Rickettsia* strains, and further studies are currently ongoing to define the role for this protein in virulence and immune activation.

## 4. Materials and Methods

### 4.1. Rickettsia Species, Strains, and Sequences

The gene and/or protein sequences used in this study were obtained from a web-based information system named Pathosystems Resource Integration Center (PATRIC) [27]. As a genomics-centric relational database and bioinformatics resource, PATRIC provides consistency in the integration and annotation of all genomic and related data for a number of bacterial pathogens, including different species and strains of all major organisms classified into ancestral, spotted fever, typhus, and transitional subgroups of rickettsiae.

### 4.2. Cell Culture

An immortalized line of human dermal microvascular endothelial cells (CDC/EU.HMEC-1) displaying all major morphologic, phenotypic, and functional features of microvascular endothelium was obtained from the Centers for Disease Control and Prevention (Atlanta, GA, USA). HMECs were maintained in continuous cultures using MCDB131 growth medium (Caisson’s Laboratories) supplemented with fetal bovine serum (10% *v*/*v*; Aleken Biologicals), epidermal growth factor (10 ng/mL, Thermo Fisher Scientific, Waltham, MA, USA), L-glutamine (10mM, Thermo Fisher Scientific, Waltham, MA, USA), and hydrocortisone (1 μg/mL, Sigma) [28,29]. All experiments were performed at the passage level of ≤35.

### 4.3. Infection

A highly virulent strain (Sheila Smith) and an avirulent strain (Iowa) of *R. rickettsii* were grown intracellularly in cultured Vero cells and purified by differential centrifugation, as described previously [30]. Rickettsial stocks were subjected to slow freezing and thawing and stored at −80 °C as aliquots of ≤500 µL to minimize repeated freeze–thaw cycles. Infectivity titers of purified stocks were estimated by citrate synthase (*gltA*)-based quantitative PCR and/or plaque-formation assay [30,31]. HMECs were infected with *R. rickettsii* at an MOI (multiplicity of infection) of approximately 20 intracellular rickettsiae per cell following our standard laboratory protocols [30,32]. The viability of both mock-infected (controls) and *R. rickettsii*-infected HMECs were monitored microscopically.

### 4.4. Phylogenetic Analysis

The phylogenetic tree and evolutionary analyses of the PleD sequences belonging to different *Rickettsia* species were performed using MEGA [33]. The PleD protein sequences were downloaded from the PATRIC database and aligned using Clustal Omega. The bootstrap consensus tree inferred from 1000 replicates was taken to represent the evolutionary history of the taxa analyzed [34]. The branches corresponding to partitions reproduced in less than 90% bootstrap replicates are collapsed. The evolutionary history was inferred using the Maximum Likelihood method based on the JTT matrix-based model [35]. Initial tree(s) for the heuristic search were obtained automatically by applying Neighbor-Join and BioNJ algorithms to a matrix of pairwise distances estimated using a JTT model, and then selecting the topology with superior log-likelihood value. The analysis involved 43 amino-acid sequences. All positions containing gaps and missing data were eliminated. There were a total of 446 positions in the final dataset. The accession numbers of PleD sequences from different *Rickettsia* species used in the analysis are provided in Table S1.

### 4.5. Protein-Structure Prediction and Modeling

The active and inactive-conformation homology models of PleD protein were constructed in MODELLER [21]. This platform builds protein models from query sequences using the solved crystal structures contained in the RCSB Protein Data Bank (PDB) as templates. The modeled structures were subjected to constrained energy minimization to allow the global energy minimization and structural analysis using the AMBER 12 suite and VADAR [36,37]. Assessment of the stereochemistry of 3D models was performed using a Ramachandran plot (PROCHECK) [38], and Protein Structure Analysis (ProSA) [39]. The active and inactive forms of PleD protein were analyzed using PyMol [40].

### 4.6. Cloning and Sequencing of Rickettsial pleD

Total genomic DNA from *R. rickettsii* strains Sheila Smith and Iowa was extracted from purified stocks using Qiagen Blood & Tissue kit (Qiagen, Germantown, MD, USA) following manufacturer’s instructions. Full-length *pleD* gene was amplified from the genomic DNA using Phusion high-fidelity taq polymerase (New England Biolabs, Ipswich, MA, USA) and using primers pleD-F (5′ CCTTTATCGCTGCCTATTTCTT 3′) and pleD-R (5′ GTGTTTTGCTGACAACCCATA 3′). The amplicon was verified by resolving on a 1.5% agarose gel and cloned into pGEM-T Easy vector (Promega, Madison, WI, USA) following manufacturer instructions. Plasmid was isolated from a positive clone carrying the *pleD* insert and was sequenced using T7 and SP6 primers at the institutional molecular genomics core facility. The resulting sequences were aligned with published sequences (NCBI Accession numbers NC_009882 (Sheila Smith) and NC_010263 (Iowa)) using Clustal Omega with default settings to identify indels and single-nucleotide polymorphisms.

### 4.7. Quantitative RT-PCR (RT-qPCR)

Total RNA was extracted from cells infected with *R. rickettsii* for different time durations and corresponding mock-infected controls was extracted using our optimized Tri Reagent ^®^ protocol [30,41]. The RNA samples were subjected to DNase I (New England Biolabs, Ipswich, MA, USA) treatment to eliminate genomic DNA contamination, precipitated using 3M sodium acetate pH 5.5 and quantified using a MultiScan™ Go Spectrophotometer (Thermo Fisher Scientific, Waltham, MA, USA). The RNA samples were then enriched for bacterial coding transcripts using MicrobEnrich™ kit for removal of eukaryotic host RNA, and MicrobExpress™ kit for removal of bacterial rRNA. The enriched samples were then assessed for purity by qRT-PCR using human *gapdh* primers (GAPDH-qPCR-F: 5′ CCACTCCTCCACCTTTGAC 3′ and GAPDH-qPCR-R: 5′ ACCCTGTTGCTGTAGCCA 3′) and only samples which exhibited a C_T_ >35 (indicating efficient removal of host transcriptome) were used for the study. Complementary DNA (cDNA) synthesis was carried out with random primers using High-Capacity cDNA Reverse Transcription kit (Applied Biosystems, Waltham, MA, USA) and absolute quantification of *pleD* expression was performed by real-time quantitative PCR. Briefly, a *pleD* amplicon was amplified using *Rickettsia*-specific primers pleD-qPCR-F (5′ TGTTATGATGCCGGAAATTGATGGA 3′) and pleD-qPCR-R (5′ AGCAGTATCGTTAATCGGCTTTGTT 3′), and cloned into a pGEM T-easy vector (Promega). The plasmid from a positive clone was then purified, linearized by restriction digestion, and used for generating the standard curve. SYBR green-assay-based absolute quantification of *pleD* expression was performed to test samples using the primers listed above.

Total DNA was extracted following our standard protocol and bacterial load in HMECs infected with *R. rickettsii* strains Sheila Smith and Iowa was estimated by citrate synthase (*gltA*)-based quantitative PCR assay, as described [30].

### 4.8. Statistical Analysis

A minimum of three independent biological replicates with two technical replicates were performed for each experiment. Statistical analysis was performed by unpaired t-test or Mann–Whitney test using GraphPad Prism software (GraphPad Software Inc., San Diego, CA, USA) and *p* value ≤ 0.05 was considered statistically significant.

## Data Availability

The nucleotide sequence of full-length *pleD* gene from *R. rickettsii* strs. Sheila Smith and Iowa sequenced in this study are deposited in the NCBI GenBank and are available under accession numbers OM714535 (*R. rickettsii* str. Sheila Smith) and OM714536 (*R. rickettsii* str. Iowa).

## Supplementary Materials

The following supporting information can be downloaded at: https://www.mdpi.com/article/10.3390/ijms23073853/s1,
