# Supplementary material for: Sensing the Messenger: Potential Roles of Cyclic-di-GMP in Rickettsial Pathogenesis
Source: Int J Mol Sci. 2022 Mar 31;23(7):3853. doi: 10.3390/ijms23073853 (PMC8999164; doi:10.3390/ijms23073853)
Supplement: Supplementary file 1 [file ijms-23-03853-s001.zip › ijms-1624694-Supplementary File S1.pdf]

Supplementary File S1: The nucleotide sequence of *pleD* gene of *Rickettsia rickettsii* strains Sheila Smith and Iowa. The full-length *pleD* gene was amplified using pleD-F and pleD-R primers as mentioned in the methods section. The PCR product was cloned into pGEM-T Easy vector and Sanger sequencing of the insert (*pleD* gene) was performed using T7 and SP6 primers (A). Alignment of *R. rickettsii* str. Iowa (Rr_I) and *R. rickettsii* str. Sheila Smith (Rr_SS) PleD protein sequences. The amino acid change at position 236 is highlighted in red (B).

A. *pleD* gene sequences from *R. rickettsii* strains

***R. rickettsii* str. Iowa**

ATGACCACAATATTAGTAGTAGATGATATAGAACCCAATATTAAGTTACTAACAGCCAAACTTTTAAAAGAGTATTA
TACGGTTCTTACTGCAAATAGCGGTAAAGAAGCACTAGCAATTCTTAAAAAAGAAAAAATTGATATTATACTACTTG
ATGTTATGATGCCGGAAATTGATGGATTTGAGGTATGTAAAACGATAAAAACCGATCCGGAAACTACTCATATACCG
GTTGTAATGGTAACTGCACTTTCCGATATTGATGATCGAGTTAAAGGTCTTGAGGCAGGAGCTGATGAGTTTTTAAC
AAAGCCGATTAACGATACTGCTCTATTTGTAAGACTTAAATCGCTATCTAGAATGAAAAGCTTAATTGATGAGTTAA
AGCTTCGTAATAGCACTAATGCATTATTAGGCGTAACAAATATCGAAATGCATGACACTTTTGCAGATAAAAAAATA
TTACTCATTAATGATGATGTGGTGCAAACTAAAAATATAAAACAAATGTTACTTAAGCTTACCCAAAACGTAAAGGT
AATAAGTAATTCCGATGAATTAGATATTATAAATGAATATACCCCCGATTTAGTAATTATCAGTAGTACGCTCGAAA
ATGACGATCCTTTGAGGATCAGTGTTATTTTAAGAGGTAAAGCAGAAATAAGCGGCGTAGTAATAATTTTACAAATT
GATGAAGACGGTATGCCTTTGGTTGTGAAAGGTATTGAACTCGGTATTAATGATTATTTCGTTTATCCTATAGAGGA
AAGCGAATTGCTTGCTAGAATTAGAACACAATTAAGACGTAAGCAATATCAAGATAATTTACGTAATGATCTTGAAC
AAAGTGTCAATTTAGCAGTTAAAGACGGTCTAACCGGCTTATTTAATCGTCGCTATTTCGATATACATCTCAAACAA
ATTATTGAGAAAACTAATAAAGAAAGTATTAAATTATGTTTACTTATGTGCGATATCGACAATTTTAAACATGTAAA
CGATACTTACGGTCATCAAGCAGGCGATAAGGTTTTAACAATTGTATCCCGTATTTTGAAGAATACTCTCAGAGTAA
CAGACTTAATAGCAAGATTCGGCGGTGAAGAATTTACTATACTCTTAACGGATATAGATATTTCTAAAGCAATTGAA
ACTGCGGAGAGAGTGAGAGTTAAAATAGAATATATGAATTTTCACATTGAAGATCAAATTGAACCTTTAAAAACAAC
TATTTCAATCGGAGTTACGAAATATAAAAAAGAAGAATCAATAGAATCTTTCATTGAACGTGCTGACAAAGCTATGT
ATGAAGCTAAAACAACGGGTAAAAATAAAGTAGTAAAATTATAA

***R. rickettsii* str. Sheila Smith**

ATGACCACAATATTAGTAGTAGATGATATAGAACCCAATATTAAGTTACTAACAGCCAAACTTTTAAAAGAGTATTA
TACGGTTCTTACTGCAAATAGCGGTAAAGAAGCACTAGCAATTCTTAAAAAAGAAAAAATTGATATTATACTACTTG
ATGTTATGATGCCGGAAATTGATGGATTTGAGGTATGTAAAACGATAAAAACCGATCCGGAAACTACTCATATACCG
GTTGTAATGGTAACTGCACTTTCCGATATTGATGATCGAGTTAAAGGTCTTGAGGCAGGAGCTGATGAGTTTTTAAC
AAAGCCGATTAACGATACTGCTCTATTTGTAAGACTTAAATCGCTATCTAGAATGAAAAGCTTAATTGATGAGTTAA
AGCTTCGTAATAGCACTAATGCATTATTAGGCGTAACAAATATCGAAATGCATGACACTTTTGCAGATAAAAAAATA
TTACTCATTAATGATGATGTGGTGCAAACTAAAAATATAAAACAAATGTTACTTAAGCTTACCCAAAACGTAAAGGT
AATAAGTAATTCCGATGAATTAGATATTATAAATGAATATACCCCCGATTTAGTAATTATCAGTAGTACGCTCGAAA
ATGACGATCCTTTGAGGATCAGTGTTATTTTAAGAGGTAAAGCAGAAATAAGCGGCGTAGTAATAATTTTACAAATT
GATGAAGACGGTATACCTTTGGTTGTGAAAGGTATTGAACTCGGTATTAATGATTATTTCGTTTATCCTATAGAGGA
AAGCGAATTGCTTGCTAGAATTAGAACACAATTAAGACGTAAGCAATATCAAGATAATTTACGTAATGATCTTGAAC
AAAGTGTCAATTTAGCAGTTAAAGACGGTCTAACCGGCTTATTTAATCGTCGCTATTTCGATATACATCTCAAACAA
ATTATTGAGAAAACTAATAAAGAAAGTATTAAATTATGTTTACTTATGTGCGATATCGACAATTTTAAACATGTAAA
CGATACTTACGGTCATCAAGCAGGCGATAAGGTTTTAACAATTGTATCCCGTATTTTGAAGAATACTCTCAGAGTAA
CAGACTTAATAGCAAGATTCGGCGGTGAAGAATTTACTATACTCTTAACGGATATAGATATTTCTAAAGCAATTGAA
ACTGCGGAGAGAGTGAGAGTTAAAATAGAATATATGAATTTTCACATTGAAGATCAAATTGAACCTTTAAAAACAAC
TATTTCAATCGGAGTTACGAAATATAAAAAAGAAGAATCAATAGAATCTTTCATTGAACGTGCTGACAAAGCTATGT
ATGAAGCTAAAACAACGGGTAAAAATAAAGTAGTAAAATTATAA

B. Alignment of PleD protein sequences from *R. rickettsii* strains

Rr_I: *R. rickettsii* str. Iowa; Rr_SS: *R. rickettsii* str. Sheila Smith

```
Rr_I        MTTILVVDDIEPNIKLLTAKLLKEYYTVLTANSGKEALAILKKEKIDIILLDVMMPEIDG     60
Rr_SS       MTTILVVDDIEPNIKLLTAKLLKEYYTVLTANSGKEALAILKKEKIDIILLDVMMPEIDG     60
            ************************************************************

Rr_I        FEVCKTIKTDPETTHIPVVMVTALSDIDDRVKGLEAGADEFLTKPINDTALFVRLKSLSR     120
Rr_SS       FEVCKTIKTDPETTHIPVVMVTALSDIDDRVKGLEAGADEFLTKPINDTALFVRLKSLSR     120
            ************************************************************

Rr_I        MKSLIDELKLRNSTNALLGVTNIEMHDTFADKKILLINDDVVQTKNIKQMLLKLTQNVKV     180
Rr_SS       MKSLIDELKLRNSTNALLGVTNIEMHDTFADKKILLINDDVVQTKNIKQMLLKLTQNVKV     180
            ************************************************************

Rr_I        ISNSDELDIINEYTPDLVIISSTLENDDPLRISVILRGKAEISGVVIILQIDEDGMPLVV     240
Rr_SS       ISNSDELDIINEYTPDLVIISSTLENDDPLRISVILRGKAEISGVVIILQIDEDGIPLVV     240
            *******************************************************:****

Rr_I        KGIELGINDYFVYPIEESELLARIRTQLRRKQYQDNLRNDLEQSVNLAVKDGLTGLFNRR     300
Rr_SS       KGIELGINDYFVYPIEESELLARIRTQLRRKQYQDNLRNDLEQSVNLAVKDGLTGLFNRR     300
            ************************************************************

Rr_I        YFDIHLKQIIEKTNKESIKLCLLMCDIDNFKHVNDTYGHQAGDKVLTIVSRILKNTLRVT     360
Rr_SS       YFDIHLKQIIEKTNKESIKLCLLMCDIDNFKHVNDTYGHQAGDKVLTIVSRILKNTLRVT     360
            ************************************************************

Rr_I        DLIARFGGEEFTILLTDIDISKAIETAERVRVKIEYMNFHIEDQIEPLKTTISIGVTKYK     420
Rr_SS       DLIARFGGEEFTILLTDIDISKAIETAERVRVKIEYMNFHIEDQIEPLKTTISIGVTKYK     420
            ************************************************************

Rr_I        KEESIESFIERADKAMYEAKTTGKNKVVKL  450
Rr_SS       KEESIESFIERADKAMYEAKTTGKNKVVKL  450
            ******************************
```
