# Supplementary material for: Sensing the Messenger: Potential Roles of Cyclic-di-GMP in Rickettsial Pathogenesis
Source: Int J Mol Sci. 2022 Mar 31;23(7):3853. doi: 10.3390/ijms23073853 (PMC8999164; doi:10.3390/ijms23073853)

Supplementary File S2: Quantification of *R. rickettsii* strains Sheila Smith and Iowa during the infection of host endothelial cells, *in vitro*. Genomic DNA from each sample was extracted at the end of the time point and rickettsial load was quantified using citrate synthase (cs) primers as described in methods. Both *R. rickettsii* str. Sheila Smith and Iowa exhibited similar levels of infection all time points and no significant differences in the rickettsial load were observed between the strains.

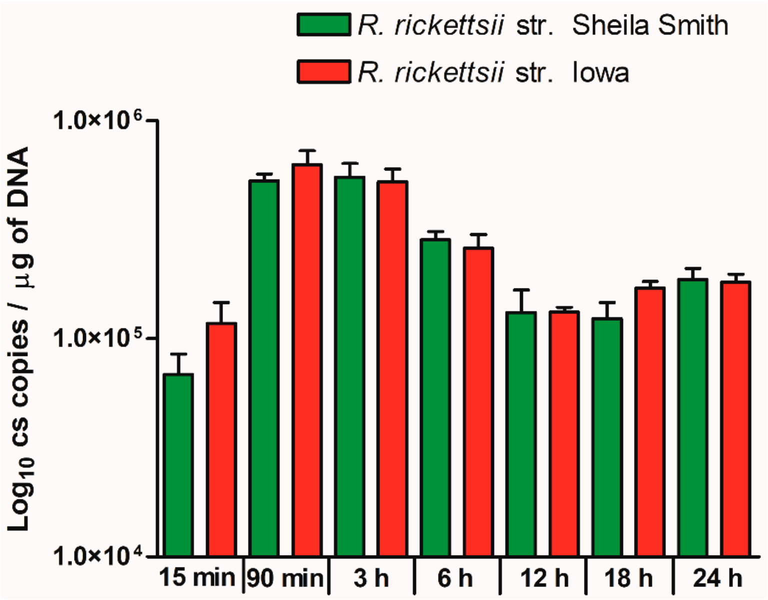

Supplement: Supplementary file 1 [file ijms-23-03853-s001.zip › ijms-1624694-Supplementary File S2.pdf]
